# Atraumatic Pyogenic Extensor Tenosynovitis of the Extensor Digitorum Longus

**DOI:** 10.7759/cureus.16952

**Published:** 2021-08-06

**Authors:** Luc M Fortier, Suhas P Dasari, Daniel B Gibbs

**Affiliations:** 1 Department of Orthopedic Surgery, Georgetown University School of Medicine, Washington, D.C., USA; 2 Department of Orthopedics, Medical College of Wisconsin, Wauwatosa, USA; 3 Department of Orthopedics, Heiden Orthopedics, Park City, USA

**Keywords:** extensor tenosynovitis, infectious tenosynovitis, tenosynovitis, septic tenosynovitis, extensor digitorum longus

## Abstract

Pyogenic tenosynovitis occurs almost exclusively in the flexor tendons of distal extremities, more commonly in the hand/wrist than the ankle/foot. Most commonly documented in the literature of the rarer extensor pyogenic tenosynovitis are case reports in the upper extremities caused by atypical bacteria or fungi, with only two cases caused by Staphylococcus aureus. It is rare for isolated tenosynovitis to occur in the extensor tendons of the lower extremity in a patient with no known trauma, IV drug use, or significant comorbidities.

We report a case of a 22-year-old male who presented with a two-day history of progressive dorsolateral foot erythema, swelling, and pain. He denied any history of trauma or evidence of foot wounds, abrasions, or punctures. His examination and ultrasound were consistent with extensor tenosynovitis of the extensor digitorum longus. He was treated with intravenous antibiotics and surgical irrigation and debridement. Intraoperatively, a large phlegmon was identified in the tenosynovium. His symptoms resolved postoperatively, and he made a full recovery with no deficits.

Pyogenic extensor tenosynovitis warrants consideration in the differential diagnosis of patients presenting with isolated dorsolateral foot erythema, swelling, and pain, despite no history of trauma or intravenous drug use.

## Introduction

Pyogenic tenosynovitis occurs almost exclusively in the flexor tendons of distal extremities, more commonly in the hand/wrist than the ankle/foot [1,2]. Most commonly documented in the literature of extensor pyogenic tenosynovitis are case reports in the upper extremities caused by atypical bacteria or fungi [3,4], with only two cases caused by Staphylococcus aureus [5,6]. In a case series of 20 patients with documented extensor pyogenic tenosynovitis, only five out of the 20 cases were in the foot/ankle region, and all of which were intravenous (IV) drug users [7]. It is rare for isolated tenosynovitis to occur in the extensor tendons of the lower extremity in a patient with no known trauma, IV drug use, or significant comorbidities. We describe a case of a 22-year-old healthy male with no known IV drug use who presented with isolated dorsolateral foot erythema, swelling, and pain found to have tenosynovitis of the extensor digitorum longus requiring antibiotic treatment and surgical irrigation and debridement.

## Case presentation

A 22-year-old male presented to the emergency department (ED) with a two-day history of slowly progressive right dorsolateral foot erythema, swelling, and pain. He denied any history of trauma to the region, changes to his shoe wear, changes in activity, or any evidence of foot wounds, abrasions, or punctures. He was encouraged to present to the emergency department by his colleagues who noticed he was limping at work. He denied any history of gout or gonococcal infection, urethral discharge, back pain, or other migratory joint pain. His past medical history was significant for bipolar two disorder, attention deficit hyperactivity disorder, binge eating disorder, chronic post-traumatic stress disorder, and generalized anxiety disorder. He denied taking any current medications. 

**Figure 1:**
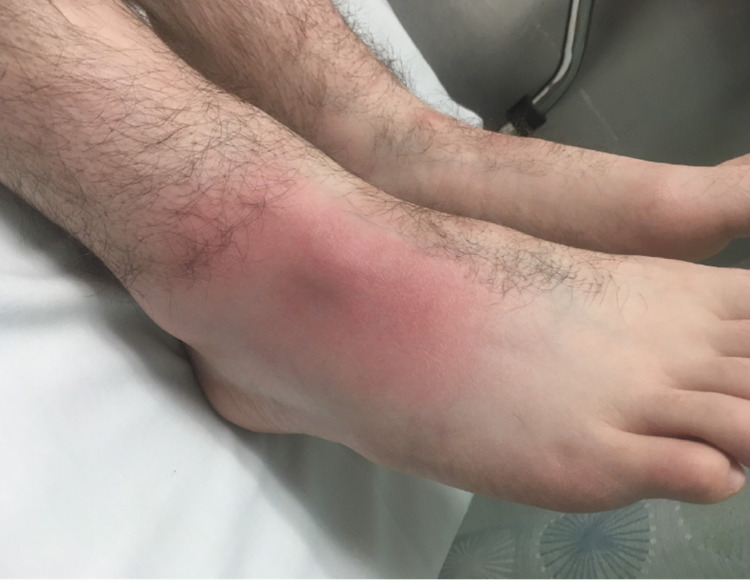
Emergency room photograph showing the right foot demonstrating an area of erythema over the dorsolateral aspect without any wounds or abrasions

On examination, his right foot demonstrated an area of erythema without any wounds or abrasions (Figure 1). 

He also demonstrated tenderness to palpation over the dorsolateral aspect without any deformity or fluctuance. He endorsed pain with passive flexion and extension of his lesser toes but no pain with passive range of motion of his ankle or subtalar joint. The patient was distally neurovascularly intact. On thorough examination, there was also no frank evidence of any trauma or abrasions elsewhere on his body. 

**Figure 2:**
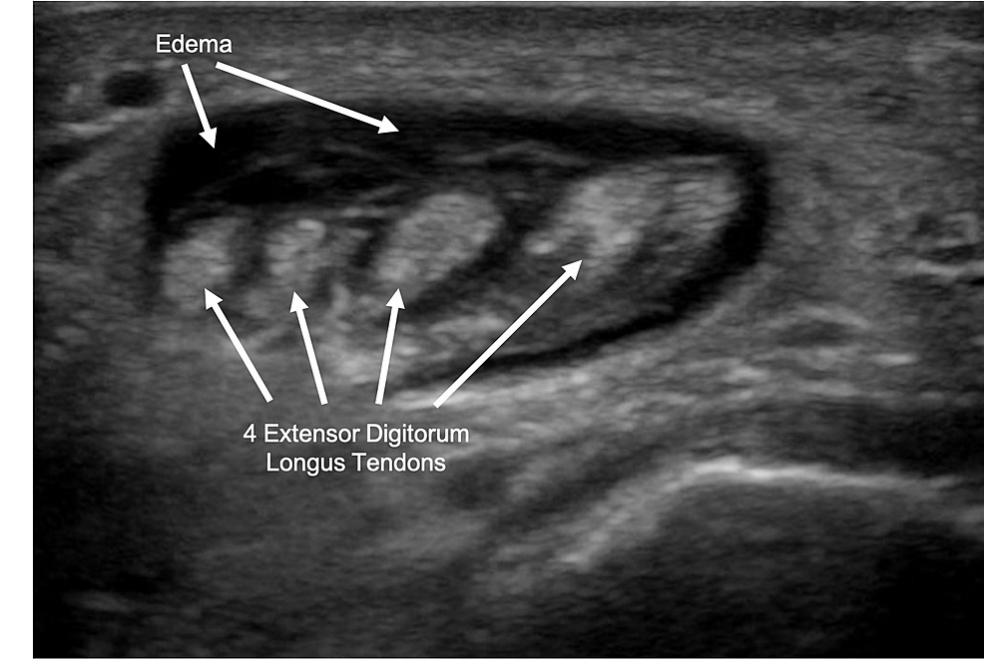
Transverse ultrasound showing soft-tissue fluid collection with hyperechoic structures in the area of extensor digitorum tendons

Prior to orthopedic evaluation, a bedside ultrasound was performed in the ED, which showed a soft-tissue fluid collection deep to the area of erythema with hyperechoic structures in the area of extensor digitorum tendons concerning tenosynovitis of the extensor digitorum longus (Figure 2).

**Figure 3:**
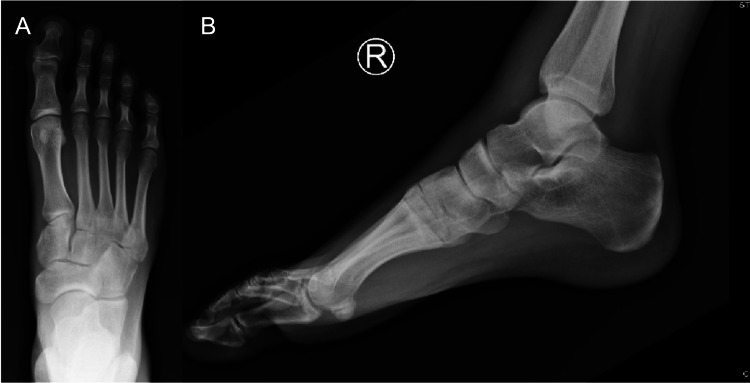
Anterior/posterior (A) and lateral (B) radiograph view of the right foot showing no evidence of fracture, gas, radiolucency, or opacity.

X-rays were negative for fracture, gas, radiolucency, or opacity (Figure 3).

His white blood cell count was elevated at 12.5 x 10^3^/$\mu$L, his c-reactive protein (CRP) was elevated at 6.1 mg/dL, and his erythrocyte sedimentation rate (ESR) was mildly elevated at 17 mm/hr. Gonorrhea and chlamydia testing were normal. No blood cultures were obtained, but broad-spectrum antibiotic treatment was initiated until intraoperative cultures could narrow the coverage. The patient was started on empiric intravenous vancomycin and ceftriaxone for the treatment of cellulitis and admitted to the hospital for further workup and management.

**Figure 4:**
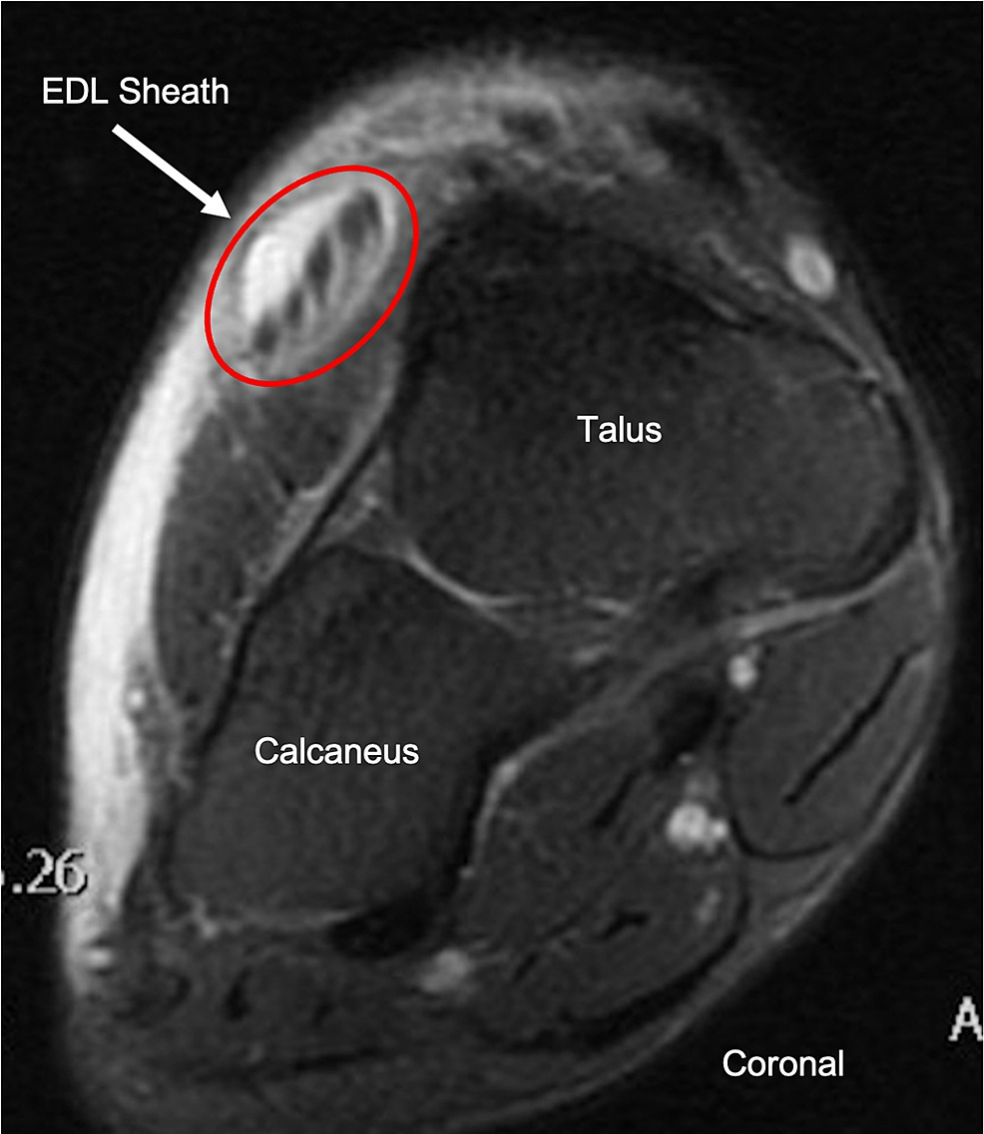
Coronal T2 FS MRI with IV gadolinium of the right foot showing dorsal soft tissue swelling and fluid within the extensor digitorum longus tendon sheath FS = fat-saturated, MRI = magnetic resonance imaging, IV = intravenous, EDL = extensor digitorum longus

Magnetic resonance imaging (MRI) the next morning demonstrated fluid within the extensor digitorum longus tendon sheath, confirming the diagnosis of tenosynovitis (Figure 4).

**Figure 5:**
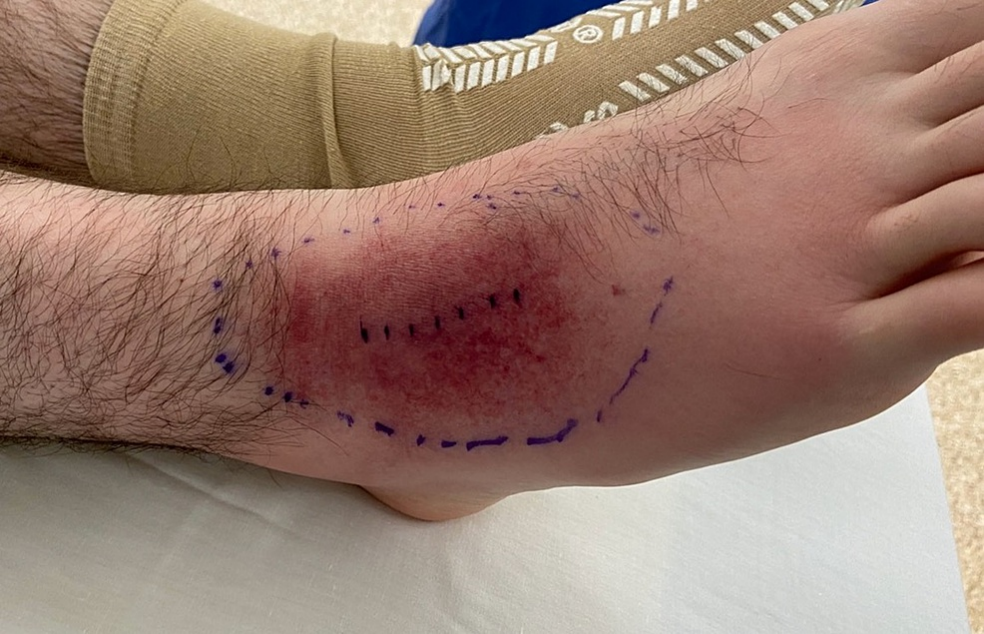
Preoperative photograph of the right foot demonstrating planned incision site in relation to margins of infection

His clinical condition did not improve over the next 24 hours, and his CRP rose to 12.8mg/dL, so the decision was made to undergo irrigation and debridement of the right foot (Figure 5). 

**Figure 6:**
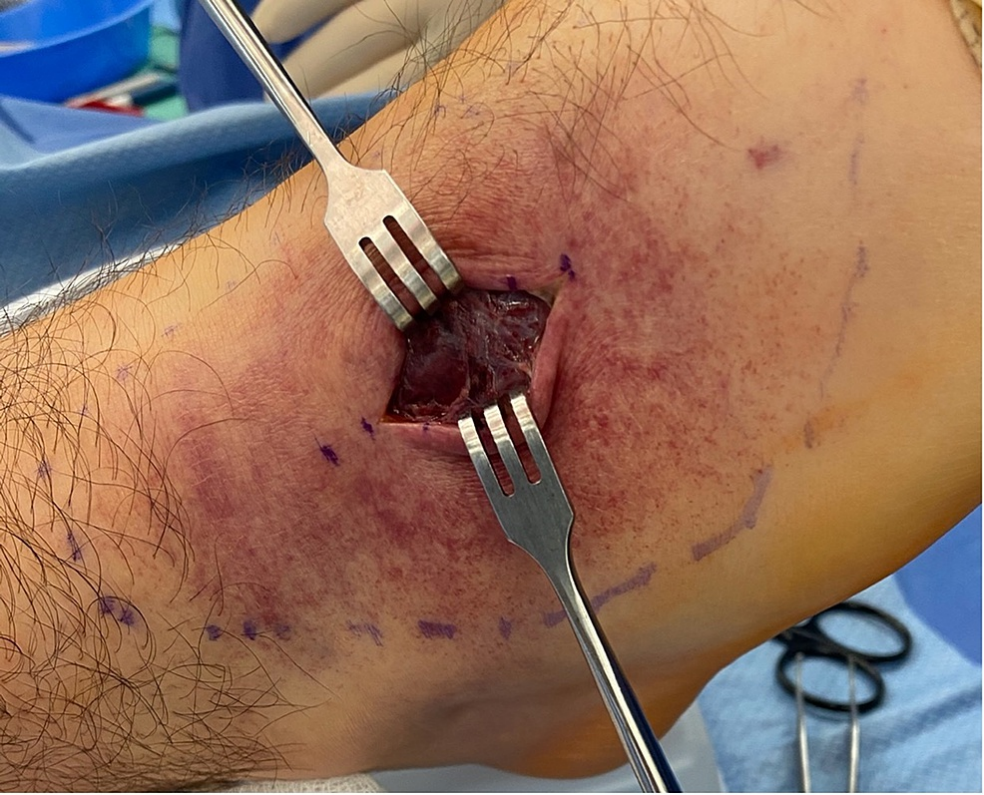
Intraoperative photograph of the right foot demonstrating the longitudinal incision and tense tenosynovium overlying the extensor digitorum longus tendons

In the operating room, a longitudinal incision was made over the dorsum of the anterolateral aspect of the right foot. The tense tenosynovium overlying the extensor digitorum longus was identified and incised in line with the incision (Figure 6).

**Figure 7:**
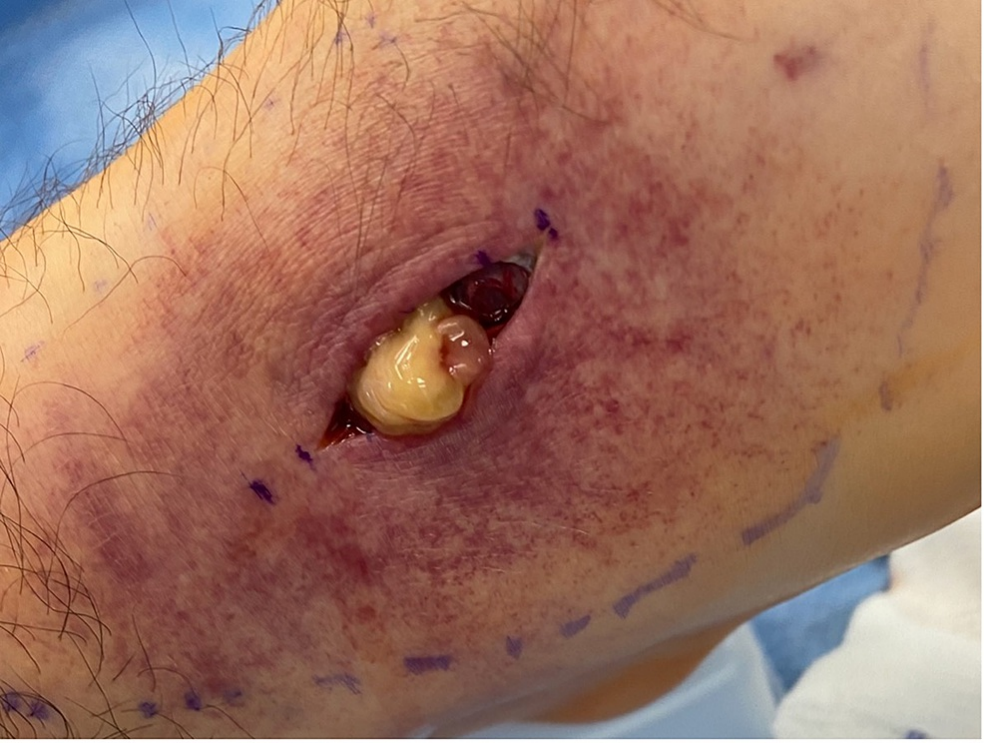
Intraoperative photograph of the exposed phlegmon present within the extensor digitorum longus tendon sheath

**Figure 8:**
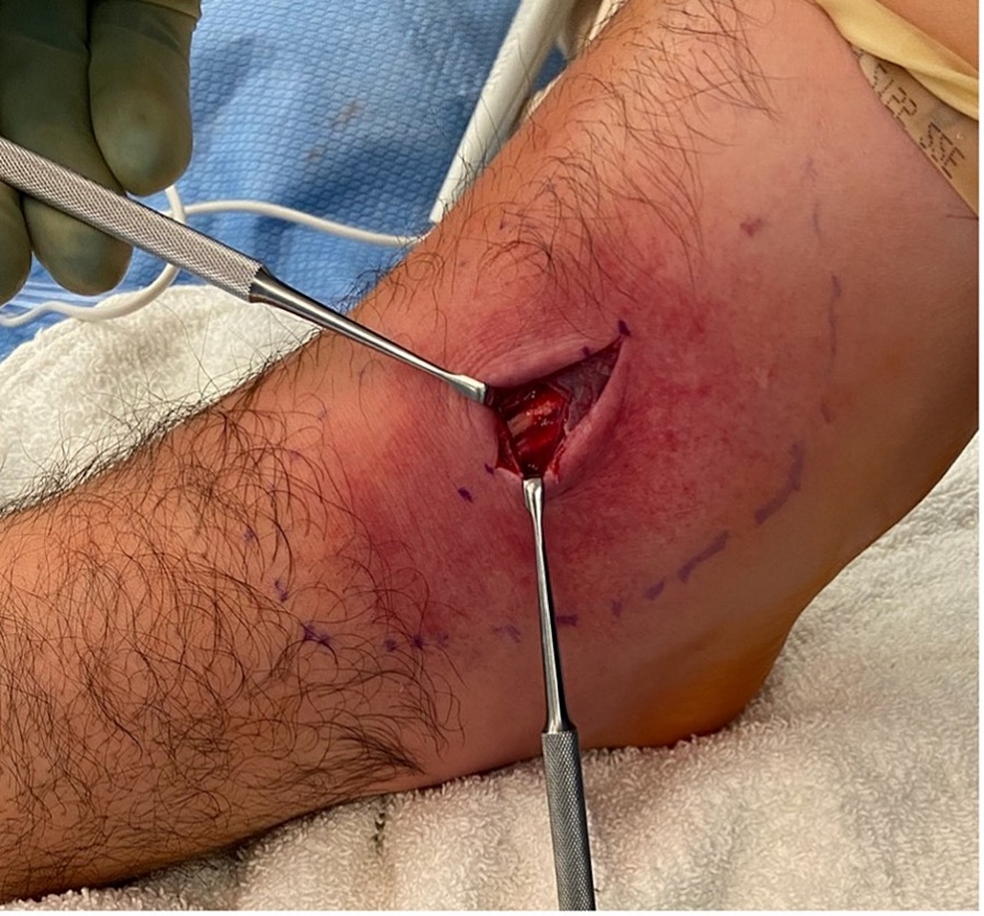
Intraoperative photograph of the right extensor digitorum longus tendon after cultures were taken

A large phlegmon was exposed (Figure 7), and cultures were taken of the phlegmon and surrounding fluid (Figure 8).

Intraoperative wound cultures were subsequently negative despite clinical evidence of infection. The wound was irrigated with 3 liters of normal saline followed by the closure of the skin. Postoperatively, the patient’s pain largely resolved, and he was discharged home postoperatively on day one with a course of oral broad-spectrum antibiotics; amoxicillin-clavulanate and doxycycline.

**Figure 9:**
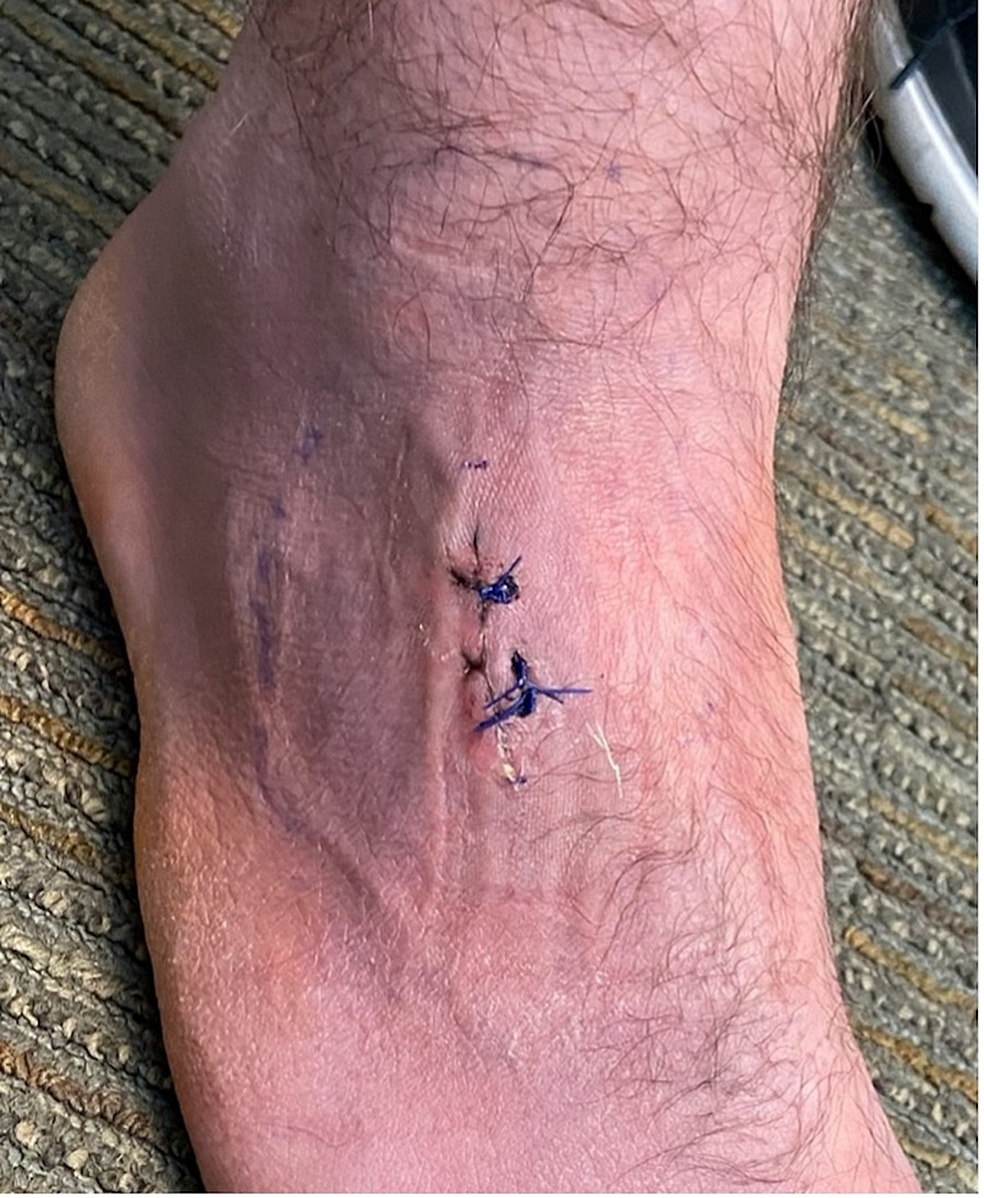
Postoperative day nine photograph of the right foot demonstrating resolution of erythema and clean, dry, and intact incision site.

At his nine-day postoperative clinic evaluation, his erythema had completely resolved, and his incision looked clean, dry, and intact (Figure 9). 

He denied any pain with passive range of motion (ROM) of his ankle, forefoot, or toes and did not have any deficits. 

## Discussion

Pyogenic tenosynovitis develops as a result of one of the three following mechanisms: trauma with direct inoculation, contiguous spread from an infection of adjacent soft tissues, or hematogenous spread [8]. Traditionally, pyogenic tenosynovitis occurs almost exclusively in the flexor tendons of distal extremities secondary to penetrating trauma, more commonly in the hand or wrist than in the foot or ankle [1,2]. A case series by Reinus et al. identified 20 cases of pyogenic extensor tenosynovitis, all of which arose from the injection site of IV drug use [7]. However, only five out of the 20 cases were identified in the foot and/or ankle region. Our case describes a rare case of a young, non-IV drug-user male with idiopathic pyogenic extensor tenosynovitis of his foot.

When pyogenic tenosynovitis presents in the foot, it usually occurs at the plantar flexors secondary to foreign body perforation [9]. Conversely, our patient denied any trauma to the region and had no evidence of abrasions, wounds, or perforations to the affected foot. Disseminated gonorrhea is another diagnosis that commonly presents with tenosynovitis in multiple joints, dermatitis with petechial or pustular skin lesions, and polyarthralgia [10]. Patients typically also present with constitutional symptoms, such as fevers and chills [11]. Testing for gonorrhea in our patient was negative.

In those rare cases without evidence of IV drug use, pyogenic extensor tenosynovitis has occurred secondary to rare fungus or atypical bacteria [3,4]. Mason et al. describe the case of a 48-year-old male with unicentric Castleman’s disease who presented with an 8-month history of progressively enlarging mass on the dorsum of his left hand [4]. He was found to have cryptococcal extensor tenosynovitis and was successfully managed with surgical debridement and antifungal therapy. Probst et al. describe another case of a 32-year-old immune-competent male who presented with chronic tuberculous-induced extensor tenosynovitis of his wrist [3]. He was treated with surgical tenosynovectomy and multidrug tuberculosis therapy. Both of these cases presented with a chronic history and described extensor tenosynovitis in the upper distal extremity. In contrast, our patient’s presentation developed acutely and in his lower extremity. To our knowledge, only two cases of acute pyogenic extensor tenosynovitis with Staphylococcus aureus have been documented in the literature, one of which blood cultures also grew Staphylococcus aureus (Table 1) [5, 6]. Both cases also presented with significant constitutional symptoms. Nevertheless, our patient’s wound and blood cultures were negative, despite evidence of purulence intraoperatively and response to antibiotic treatment.

**Table 1:** Available literature describing documented cases of pyogenic extensor tenosynovitis

Author (Year)	Location	Study Design	Associations	Microbiology	Treatment
Mason (2011) [4]	Dorsal extensor compartment of the wrist	Case Report	Castleman’s disease	Cryptococcus neoformans	Surgical debridement & antifungal therapy
Probst (2012) [3]	Dorsal extensor compartment of the wrist	Case Report	Immunocompromised	Tuberculosis	Surgical debridement & multidrug TB therapy
Reinus (2014) [7]	Wrist/hand (n=15) Ankle/foot (n=5)	Case Series	IV drug use	MSSA, MRSA, B-hemolytic streptococcus, polymicrobial	Surgical debridement & antibiotic therapy tailored to microorganism
Guillen (2014) [6]	Foot extensor tendons	Case Report	n/a	Staphylococcus aureus	Surgical debridement & vancomycin antibiotic therapy
Newman (1989) [5]	Extensor tendons of the thumb	Case Report	Diabetes mellitus, cervical cancer, recurrent pyocystitis	Staphylococcus aureus	Surgical debridement & cefazolin/gentamicin antibiotic therapy

In contrast to flexor tendons, most extensor tendons lack an extensive, isolated retinacular system making them less loculated. Consequently, extensor tenosynovitis may mimic a simple soft tissue infection and delay early diagnosis [12]. If left untreated, increased pressure within the sheath may lead to tendon necrosis [13]. Other complications include subcutaneous purulence secondary to tendon sheath rupture and cutaneous signs of ischemia. Less commonly, these infections may lead to osteomyelitis or necrotizing fasciitis [8]. A review by Giladi, although discussing acute pyogenic flexor tenosynovitis, concluded that early administration of antimicrobial therapy, prompt irrigation, and, if necessary, debridement leads to a better prognosis [14]. Fortunately, ultrasonography was promptly performed on our patient’s foot on presentation to the emergency department to rule out fluid collection or an abscess. The findings of fluid surrounding the area of the extensor digitorum tendons raised concern for extensor tenosynovitis, which resulted in the early administration of IV antibiotics and admission to the floor for further workup and management. He was successfully treated with surgical irrigation and debridement within 36 hours of admission and discharged the following day.

## Conclusions

This case demonstrates that prompt recognition, early antibiotic treatment, and surgical debridement is effective in treating idiopathic extensor tenosynovitis of the EDL. Pyogenic tenosynovitis of the extensor tendon sheaths is rarely reported and even more uncommon in the distal lower extremities, especially with no evidence of penetrating trauma. This case demonstrates pyogenic extensor tenosynovitis can occur in young males without any significant co-morbidities, constitutional symptoms, or history of IV drug use or trauma. 

Pyogenic tenosynovitis diagnosis should be considered when clinicians encounter patients presenting with isolated dorsal foot erythema, swelling, and pain. Early recognition of this rare presentation may avoid unnecessary morbidity and improved prognosis.
